# Complement activation contributes to perioperative neurocognitive disorders in mice

**DOI:** 10.1186/s12974-018-1292-4

**Published:** 2018-09-04

**Authors:** Chao Xiong, Jinhu Liu, Dandan Lin, Juxia Zhang, Niccolò Terrando, Anshi Wu

**Affiliations:** 1 Department of Anesthesiology, Beijing Chao-Yang Hospital, Capital Medical University, Beijing, 100020 China; 2 Department of Anesthesiology, Beijing First Hospital of Integrated Chinese and Western Medicine, Beijing, 100021 China; 3 Center for Translational Pain Medicine, Department of Anesthesiology, Duke University Medical Center, Durham, NC 27710 USA

**Keywords:** Choroid plexus, Complement, Hippocampus, Neuroinflammation, Perioperative neurocognitive disorders

## Abstract

**Background:**

The complement system plays an important role in many neurological disorders.

Complement modulation, including C3/C3a receptor signaling, shows promising therapeutic effects on cognition and neurodegeneration. Yet, the implications for this pathway in perioperative neurocognitive disorders (PND) are not well established. Here, we evaluated the possible role for C3/C3a receptor signaling after orthopedic surgery using an established mouse model of PND.

**Methods:**

A stabilized tibial fracture surgery was performed in adult male C57BL/6 mice under general anesthesia and analgesia to induce PND-like behavior. Complement activation was assessed in the hippocampus and choroid plexus. Changes in hippocampal neuroinflammation, synapse numbers, choroidal blood-cerebrospinal fluid barrier (BCSFB) integrity, and hippocampal-dependent memory function were evaluated after surgery and treatment with a C3a receptor blocker.

**Results:**

C3 levels and C3a receptor expression were specifically increased in hippocampal astrocytes and microglia after surgery. Surgery-induced neuroinflammation and synapse loss in the hippocampus were attenuated by C3a receptor blockade. Choroidal BCSFB dysfunction occurred 1 day after surgery and was attenuated by C3a receptor blockade. Administration of exogenous C3a exacerbated cognitive decline after surgery, whereas C3a receptor blockade improved hippocampal-dependent memory function.

**Conclusions:**

Orthopedic surgery activates complement signaling. C3a receptor blockade may be therapeutically beneficial to attenuate neuroinflammation and PND.

**Electronic supplementary material:**

The online version of this article (10.1186/s12974-018-1292-4) contains supplementary material, which is available to authorized users.

## Background

Cognitive impairments are common problems especially amongst older surgical patients [1]. These neurological complications, termed as perioperative neurocognitive disorders (PND) [2], associate with poor functional recovery and increased mortality after major surgery [3]. Although the pathogenesis of PND remains unclear, preclinical studies suggest that surgery triggers acute systemic inflammation [4] followed by neuroinflammation [5–7] and synaptic dysfunction [8, 9], which appear to contribute to hippocampal-dependent cognitive deficits. Recent human studies describe similar pathological hallmarks after major surgery including biomarkers of systemic inflammation, neuroinflammation, and neuronal damage [10, 11]. Strategies aimed at modulating this immune response have shown promising effects in animal models; however, no effective strategies for the treatment and/or prevention of PND are available for clinical use yet.

The complement system is well-known to play an important role in innate immunity regulation [12]. Emerging evidence shows that the complement system also serves many pivotal functions in the central nervous system (CNS) [13, 14]. Under homeostatic conditions, complement pathways help eliminating cellular debris, apoptotic cells, and pathogens [13], as well as regulating synaptic pruning during brain development [15]. In contrast, abnormal activation of the complement system has been related to several CNS pathologies and neurodegenerative conditions [16, 17].

The central component of the complement system, C3, has been extensively investigated in the CNS [16–21]. C3a, a cleavage product of C3, binds to the G protein-coupled receptor named C3a receptor (C3aR) [22]. Both pharmacological blockade and genetic deficiency in C3aR have therapeutic effects in models of neuroinflammation, synapse loss, and cognitive dysfunction in rodents [17, 19, 20]. Herein, we hypothesize that orthopedic surgery induces C3/C3aR signaling activation in the CNS, thus contributing to PND pathogenesis. Further, we demonstrate that administration of C3a can trigger neuroinflammation whereas C3aR blockade provides therapeutic benefits that may inform about novel clinical trials.

## Methods

### Mice

All experiments were approved by Institutional Animal Care and Use Committee at Capital Medical University (Beijing, China) and performed under the regulations of Medical Research Center of Beijing Chao-Yang Hospital (Beijing, China). Twelve- to 14-week-old male C57BL/6 mice were purchased from Vital River Laboratory (Beijing, China). All mice were housed under a 12 h light/dark cycle with free access to food and water in the vivarium of Beijing Chao-Yang Hospital.

### Experimental design

**Fig. 1:**
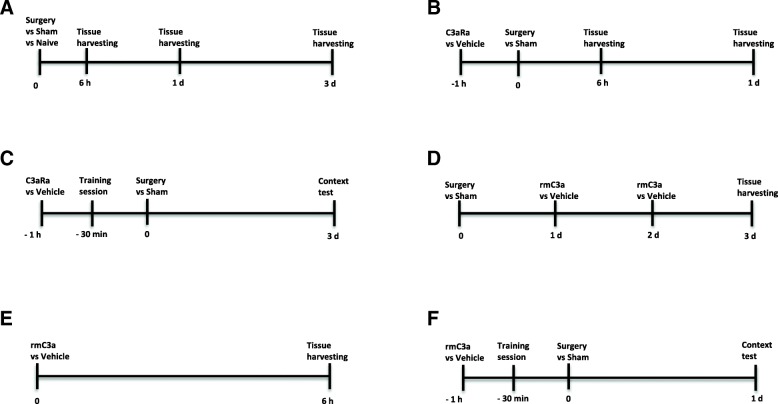
Study design. **a** Mice were randomly assigned to three groups: naive, sham, and surgery. Mice were sacrificed for tissues harvesting at 6 h, 1 day, and 3 days after surgery or sham. **b** Mice were randomly assigned to three groups: sham + vehicle, surgery + vehicle, and surgery + C3aR antagonist (C3aRa). C3aRa or vehicle was given 1 h prior to surgery or sham. Mice were sacrificed for tissues harvesting at 6 h and 1 day after surgery or sham. **c** Mice were randomly assigned to three groups: sham + vehicle, surgery + vehicle, and surgery + C3aR antagonist (C3aRa). 30 min after C3aRa or vehicle administration, mice were subjected to the training session for trace fear conditioning. Mice underwent surgery or sham 30 min after training. At 3 days after surgery or sham, context test was performed. **d** Mice were randomly assigned to three groups: sham + vehicle, surgery + vehicle, and surgery + recombinant mouse C3a (rmC3a). Mice were given intranasal rmC3a or vehicle at 24 h and 48 h after surgery or sham procedure. Mice were sacrificed for tissues harvesting at 3 days. **e** Naïve mice were randomly assigned to vehicle or rmC3a treatment; at 6 h after intranasal rmC3a or vehicle administration, mice were sacrificed for tissue harvesting. **f** Mice were randomly assigned to three groups: sham + vehicle, surgery + vehicle, and surgery + rmC3a. 30 min after rmC3a or vehicle administration, mice were trained for trace fear conditioning. Mice underwent surgery or sham 30 min after training. The context test was performed at 1 day after surgery or sham procedure

The study design is presented in Fig. 1.

### Surgery

Orthopedic surgery was performed as previously described [23]. Briefly, mice received an open tibia fracture with intramedullary pinning under 2% isoflurane anesthesia. Buprenorphine (0.1 mg/kg) was administered subcutaneously after anesthesia induction. Sham mice underwent exactly the same anesthesia and analgesia but without surgical intervention.

### Intraperitoneal administration of C3a receptor antagonist

C3aR antagonist (C3aRa) (Millipore, #559410) was dissolved in phosphate-buffered saline (PBS) containing 0.5% dimethyl sulfoxide (DMSO). 1 h before surgery, mice were intraperitoneally administered C3aR antagonist (1 mg/kg, IP). Vehicle-treated mice received IP administration of 0.5% DMSO in PBS. This antagonist has been extensively used for C3aR blockade in treating CNS disorders in rodents [17, 19, 21].

### Intranasal administration of recombinant mouse C3a

Intranasal drug administration was performed as previously described [24] with minor modifications. Mice were acclimated for handling to minimize stress response before drug administration. Recombinant mouse C3a (rmC3a) (10 μg/kg, R&D systems, #8085-C3-025) was dissolved in PBS and intranasally given to each restrained awake mouse in a total volume of 10 μL. Drugs were ejected as small droplets using a pipettor and inhaled through the mouse’s nostril. Vehicle-treated mice were given an equal volume of PBS. Timepoints for intranasal C3a delivery in each experiment were described in Fig. 1d–f. This intranasal approach has been successfully used to deliver exogenous C3a to the mouse brain in previous studies [25, 26].

### Enzyme-linked immunosorbent assay

Mice were transcardially perfused with ice cold PBS. Hippocampal tissues were harvested and homogenized. Collected supernatants were quantified by bicinchoninic acid (BCA) assay (Thermo Scientific, #23225). Enzyme-linked immunosorbent assays (ELISA) were performed to detect hippocampal levels of C3, interleukin-1β (IL-1β), and interleukin-6 (IL-6) using commercially available ELISA kits (Abcam, #ab157711; R&D Systems, #MLB00C; Thermo Scientific, #KMC0061).

### Western blotting

Homogenized hippocampal tissues were quantified by BCA assay. Denatured proteins were separated by 10% sodium dodecyl sulfatepolyacrylamide gel electrophoresis (Bio-Rad) and transferred onto polyvinylidene fluoride membranes (Thermo Scientific). Membranes were blocked for 1 h at room temperature (RT) and subsequently incubated at 4 °C overnight with primary antibodies against synaptophysin (SYP) (1:1000, Sigma, #S5768), postsynaptic density protein 95 (PSD-95) (1:1000, Cell Signaling, #2507), or beta-actin (1:1000, Thermo Scientific, # MA5-15739). After washing, membranes were incubated with horseradish peroxidase (HRP) conjugated secondary antibodies (1:5000, Thermo Scientific) at RT for 1 h. Finally, membranes were incubated with chemiluminescent HRP substrate (Thermo Scientific) and imaged by ChemiDoc XRS^+^ system (Bio-Rad). The intensity values of target bands were measured by Image Lab software (Bio-Rad).

### Immunohistochemistry

Mice were perfused with ice-cold PBS containing 4% paraformaldehyde (PFA). Harvested brains were post-fixed with 4% PFA in PBS for 24 h at 4 °C. After washing with PBS, brains were immersed in 30% sucrose/PBS solution for 48 h at 4 °C. A freezing microtome was used to cut brains into 30-μm-thick free-floating sections. The sections were blocked with 1% bovine serum albumin plus 0.2% Triton X-100 (Sigma, #T8787) in PBS for 1 h at RT. After blocking, the sections were incubated with primary antibodies against C3 (1:100, Abcam, #ab11862), C3aR (1:100, Hycult Biotech, #HM1123), ionized calcium-binding adapter molecule 1 (IBA1) (1:500, Wako, #019-19741), glial fibrillary acidic protein (GFAP) (1:1000, Dako, #Z0334), CD68 (1:200, Bio-Rad, #MCA1957), intercellular adhesion molecule-1 (ICAM-1) (1:200, R&D Systems, #AF796), vascular cell adhesion molecule-1 (VCAM-1) (1:100, Abcam, #134047), myeloperoxidase (MPO (1:100, Abcam, #ab9535), or mouse immunoglobulin G (IgG) (1:200, Invitrogen, #A-21203) overnight at 4 °C. After washing with PBS, the sections were incubated with fluorophore-conjugated secondary antibodies (1:200, Invitrogen) at RT for 2 h. The sections were washed again with PBS, mounted on slides, and sealed with Fluoroshield mounting medium (Sigma, #F6057) and 0.17-mm-thick coverslips. Images were acquired using a Leica SP8 confocal microscope and then processed by Adobe Photoshop software (version CS6). Images from different experimental groups were captured and adjusted under the same conditions. Quantitative analyses were done using ImageJ software (version 2.00).

### Trace fear conditioning

Trace fear conditioning has been widely used for assessing hippocampal-dependent memory in PND [4]. 30 min after C3aR antagonist, rmC3a, or vehicle administration, mice received a training session to associate a conditional stimulus (context) with an unconditional stimulus (two periods of 2-s foot-shocks of 0.75 mA each). 30 min after training, mice were subjected to tibia fracture or sham surgery. One or 3 days after training, mice were tested in the same context but received no unconditional stimulus (foot shocks). Freezing behavior for each mouse was recorded and analyzed by a camera-based monitoring system (Xeye Fcs system, Beijing MacroAmbition S&T Development Co., Ltd., Beijing, China).

### Statistics

Statistical analysis was performed with GraphPad Prism V6 (GraphPad Software, La Jolla, CA). Comparisons between different groups were made using one-way analysis of variance (ANOVA) with repeated measures followed by Tukey’s or Student-Newman-Keuls test. Unpaired Student’s *t* test was used for comparisons between two groups. Relationships between two variables were evaluated using linear regression. Statistical significance was indicated when *p* < 0.05. Data are means ± standard error of the mean.

## Results

### Orthopedic surgery induces astrocytic C3 and microglial C3aR upregulation in the hippocampus

**Fig. 2:**
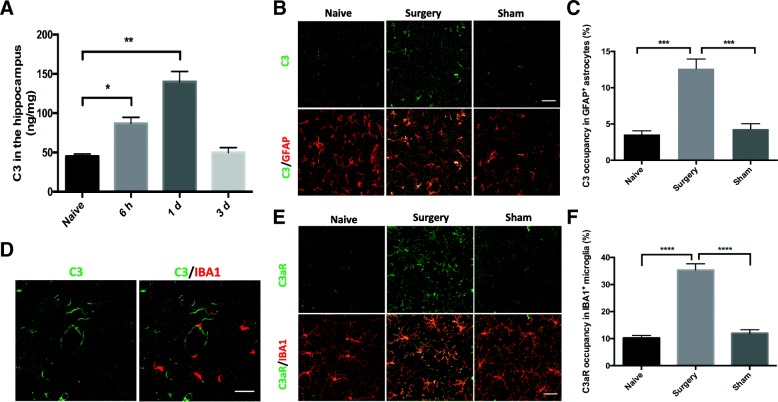
Orthopedic surgery induces complement activation in the hippocampus. **a** C3 was assayed by ELISA in hippocampal homogenates of naïve and surgery mice (one-way analysis of variance followed by Student-Newman-Keuls test; *n* = 5). **b** Representative confocal images of C3 (green) and astrocytic marker GFAP (red) immunostaining in the hippocampus of naïve, surgery, and sham mice on postoperative day 1. **c** Quantification of C3 occupancy in GFAP^+^ astrocytes (one-way analysis of variance followed by Tukey post hoc test; *n* = 5). **d** Representative confocal images of C3 (green) and microglial marker IBA1 (red) double immunostaining in the hippocampus 1 day after surgery. **e** Representative confocal images of C3aR (green) and IBA1 (red) labeling in the hippocampus of naïve, surgery, and sham mice at 1 day. **f** Quantification of C3aR occupancy in IBA1^+^ microglia, normalized to the level in the naïve group (one-way analysis of variance followed by Tukey post hoc test; *n* = 5). Scale bar = 30 μm (**b**, **d**, and **e**). *p < 0.05, **p < 0.01, ***p < 0.001, ****p < 0.0001

To determine whether major surgery activates the complement system we assessed the level of the central complement component, C3, in the hippocampus after orthopedic surgery. Hippocampal C3 was elevated at 6 h (87.16 ± 7.35 ng/mg, *p* < 0.05; Fig. 2a), peaking on postoperative day 1 (140.20 ± 12.68 ng/mg, *p* < 0.01; Fig. 2a), and returning to baseline by postoperative day 3 (49.95 ± 6.29 ng/mg vs. 45.30 ± 2.74 ng/mg, *p* > 0.05; Fig. 2a).

Next, we interrogated the cellular distribution of C3 in the hippocampus focusing on three cell types: astrocytes, microglia, and neurons on postoperative day 1, the peak of surgery-induced C3 elevation in this model. Compared to the naïve group, C3 was found significantly elevated in GFAP^+^ astrocytes after surgery (surgery vs. naïve: *p* < 0.001; Fig. 2b, c) but was absent in neurons (Additional file 1). Notably, we found no co-localization of C3 with the microglial marker IBA1 (Fig. 2d); however, IBA1^+^ cells expressed higher C3aR after surgery (surgery vs. naïve: *p* < 0.0001; Fig. 2e, f). In contrast, sham surgery did not trigger either astrocytic C3 (sham vs. naïve: *p* > 0.05; Fig. 2b, c) or microglial C3aR upregulation (sham vs. naïve: *p* > 0.05; Fig. 2e, f).

### Orthopedic surgery-induced neuroinflammation is attenuated by C3aR blockade

To illustrate the role of C3/C3aR signaling in surgery-induced neuroinflammation, we examined the effects of a selective C3aR antagonist on pro-inflammatory cytokines, microglial activation, and neutrophil infiltration in the hippocampus.

**Fig. 3:**
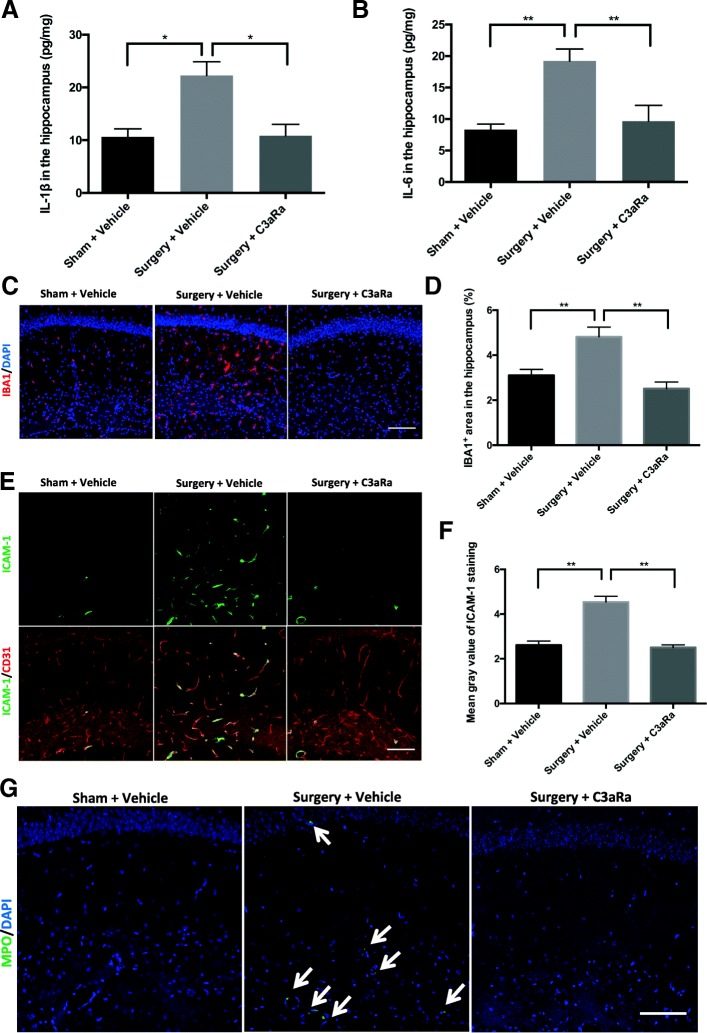
C3aR blockade ameliorates surgery-induced neuroinflammation. Mice were randomly assigned to three groups (*n* = 5/group): sham + vehicle, surgery + vehicle, and surgery + C3aR antagonist (C3aRa). ELISA was performed to assay proinflammatory cytokines IL-1β (**a**) and IL-6 (**b**) in hippocampal homogenates at 6 h after surgery or sham procedure. **c** Representative confocal images of IBA1 (red) labeling in the hippocampus at 1 day after surgery or sham procedure. **d** Quantification of percentage of IBA1^+^ area. **e** Double immunostaining of adhesion molecular ICAM-1 (green) and endothelial marker CD31 (red) in the hippocampus. **f** Quantification of ICAM-1 by relative fluorescence intensity. **g** Representative images of anti-myeloperoxidase (MPO) (green) immunostaining; white arrows indicate MPO^+^ cells. Nuclear counterstaining with DAPI (blue) (**c**, **g**). Scale bar = 100 μm (**c**, **e**, and **g**). Data analyses were performed using one-way analysis of variance followed by Tukey post hoc test; *p < 0.05, **p < 0.01

Pro-inflammatory cytokines IL-1β and IL-6 in the hippocampus were measured using ELISA. Compared to the sham group, orthopedic surgery increased both IL-1β (surgery + vehicle vs. sham + vehicle: 22.30 ± 2.58 pg/mg vs. 10.64 ± 1.52 pg/mg, respectively, *p* < 0.05; Fig. 3a) and IL-6 (surgery + vehicle vs. sham + vehicle: 19.22 ± 1.895 pg/mg vs. 8.33 ± 0.86 pg/mg, respectively, *p* < 0.01; Fig. 3) at 6 h. In contrast, treatment with the C3aR antagonist abolished the upregulation of hippocampal IL-1β (surgery + C3aRa vs. surgery + vehicle: 10.87 ± 2.15 pg/mg 22.30 ± 2.58 pg/mg, respectively, *p* < 0.05; Fig. 3a) and IL-6 (surgery + C3aRa vs. surgery + vehicle: 9.67 ± 2.50 pg/mg vs. 19.22 ± 1.90 pg/mg, respectively, *p* < 0.01, Fig. 3b).

Microglial activation was evaluated by IBA1 immunoreactivity in the hippocampus. Compared to sham mice, orthopedic surgery caused significant increase in IBA1 expression (surgery + vehicle vs. sham + vehicle: *p* < 0.01; Fig. 3c, d) on day 1 as previously shown in this model [23]. In contrast, C3aR blockade ameliorated hippocampal microglial activation after surgery (surgery + C3aRa vs. surgery + vehicle: *p* < 0.01; Fig. 3c, d).

Next, we evaluated hippocampal neutrophil infiltration using ICAM-1 and MPO immunostainings. ICAM-1, a well-characterized adhesive molecule that mediates leukocyte trafficking [27], was significantly elevated after surgery (surgery + vehicle vs. sham + vehicle: *p* < 0.01; Fig. 3e, f) and co-localized with the endothelial cell marker CD31 (Fig. 3e). Notably, ICAM-1 elevation was reversed by C3aR antagonist treatment (surgery + C3aRa vs. surgery + vehicle: *p* < 0.01; Fig. 3e, f). Orthopedic surgery also induced infiltration of MPO^+^ neutrophils in the hippocampus (Fig. 3g), which was blocked by C3aR antagonist (Fig. 3g).

### C3aR antagonist reduces microglial phagocytic activity and synapse loss after orthopedic surgery

**Fig. 4:**
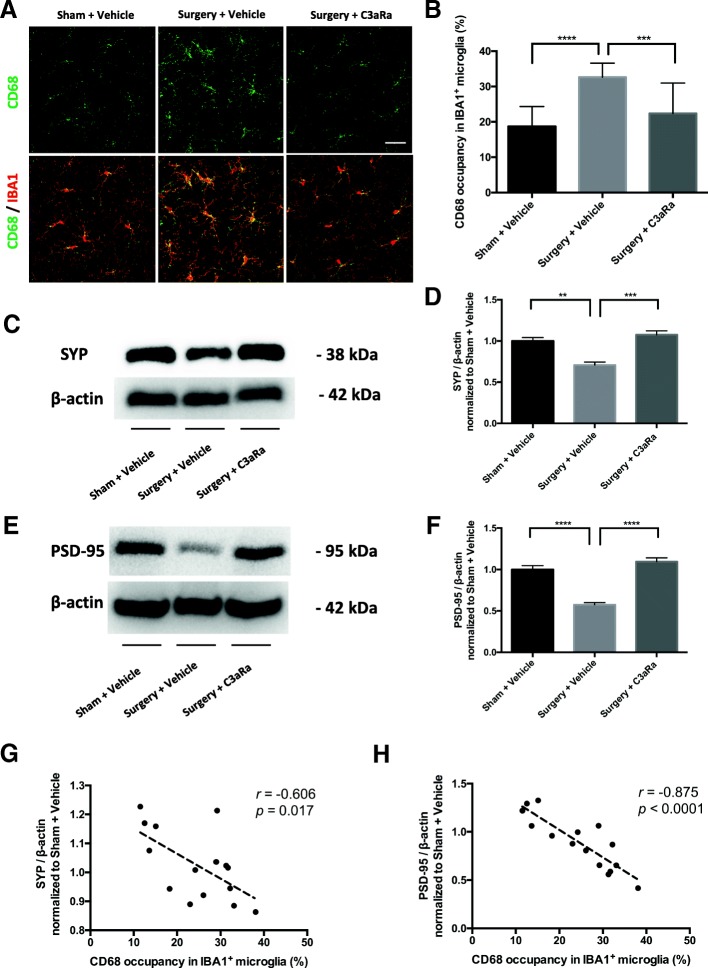
C3aR blockade reduces microglial phagocytic activity and synapse loss at 1 day after orthopedic surgery. Mice were randomly assigned to three groups (*n* = 5/group): sham + vehicle, surgery + vehicle, and surgery + C3aR antagonist (C3aRa). **a** Representative confocal images of double immunostaining of CD68 (green) and IBA1 (red); scale bar = 30 μm. **b** Quantification of CD68 occupancy in IBA1^+^ microglia. Representative images from western blotting of presynaptic marker SYP (**c**) and postsynaptic marker PSD-95 (**e**). Quantification of SYP (**d**) and PSD-95 (**f**). Linear regression analyses of the relationship between microglia CD68 reactivity and synaptic marker SYP (**g**) or PSD-95 (**h**). Data analyses were performed using one-way analysis of variance followed by Tukey post hoc test (**b**, **d**, **f**) or regression analysis (**g**, **h**). **p < 0.01, ***p < 0.001, ****p < 0.0001

Complement-induced synapse loss has been implicated in the development of cognitive impairment [16, 20]. To evaluate whether orthopedic surgery-induced C3/C3aR signaling activation contributes to synaptic dysfunction we assessed the effects of C3aR blockade on microglial phagocytic activity and synapse loss. Orthopedic surgery increased microglial phagocytic activity as measured by CD68 at 1 day postoperatively (surgery + vehicle vs. sham + vehicle: *p* < 0.0001; Fig. 4a, b). Notably, surgery-induced microglial CD68 upregulation was attenuated by C3aR antagonist (surgery + C3aRa vs. surgery + vehicle: *p* < 0.001; Fig. 4a, b). To further evaluate the effects on synapse numbers, we measured hippocampal levels of the presynaptic protein SYP and postsynaptic protein PSD-95. Orthopedic surgery reduced both SYP (surgery + vehicle vs. sham + vehicle: 0.71 ± 0.04 vs. 1.00 ± 0.04, *p* < 0.01; Fig. 4c, d) and PSD-95 (surgery + vehicle vs. sham + vehicle: 0.57 ± 0.03 vs. 1.00 ± 0.05, *p* < 0.0001; Fig. 4e, f). In contrast, the C3aR antagonist improved surgery-induced reduction in SYP (surgery + C3aRa vs. surgery + vehicle: 1.08 ± 0.05 vs. 0.71 ± 0.04, *p* < 0.001; Fig. 4c, d) and PSD-95 (surgery + C3aRa vs. surgery + vehicle: 1.09 ± 0.05 vs. 0.57 ± 0.03, *p* < 0.0001; Fig. 4e, f).

Notably, upregulation of microglial phagocytic activity has been related to increased synapse engulfment [16, 20, 28]. Here, we showed a significant correlation between microglial CD68 expression and synapse numbers (SYP: *r* = − 0.606, *p* = 0.017, Fig. 4g; PSD-95: *r* = − 0.875, *p* < 0.0001, Fig. 4h), suggesting that microglial phagocytic activity associates with synapse loss in the hippocampus after surgery.

### C3aR blockade attenuates blood-CSF barrier dysfunction in the choroid plexus after orthopedic surgery

**Fig. 5:**
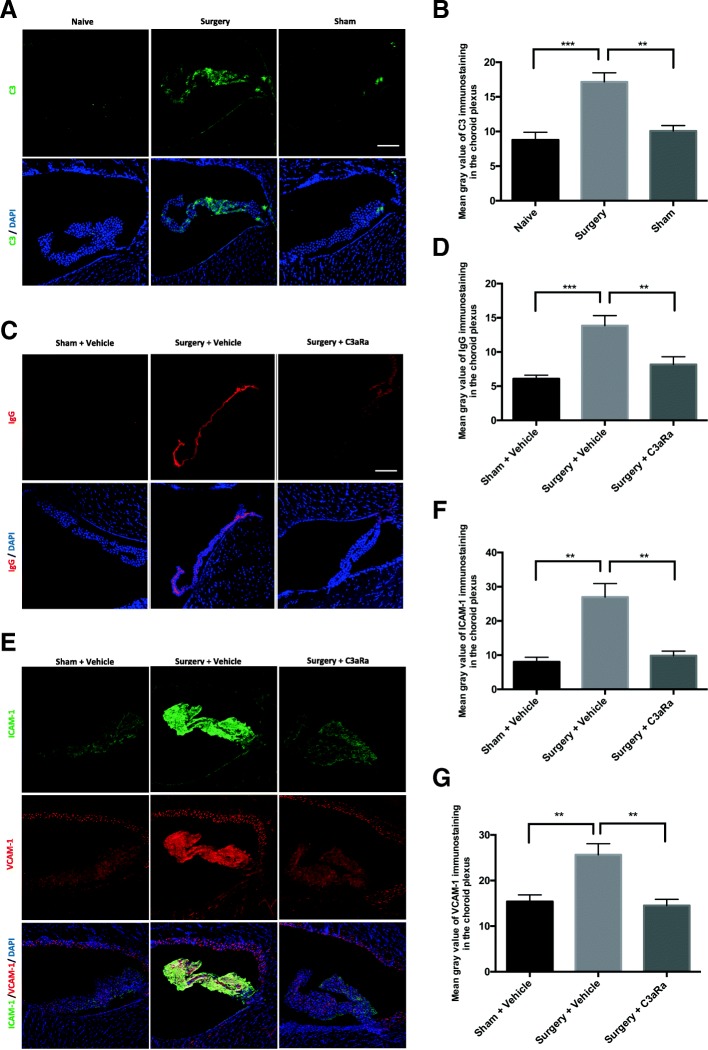
C3aR blockade attenuates BCSFB disruption in the choroid plexus after surgery. **a** Representative images of C3 labeling in the choroid plexus of naïve, surgery, and sham mice at 1 day after surgery or sham. **b** Quantification of C3 fluorescence intensity. **c** Representative images of IgG staining in the choroid plexus of 3 groups: sham + vehicle, surgery + vehicle, and surgery + C3aRa. **d** Quantification of IgG fluorescence intensity. **e** Representative images of ICAM-1 (green) and VCAM-1 (red) labelings in the choroid plexus. Quantification of ICAM-1 (**f**) and VCAM-1 (**g**) fluorescence intensity. Nuclear counterstaining with DAPI (blue) (**a**, **c**, and **e**). Scale bar = 100 μm (**a**, **c**, and **e**). Data analyses were performed using one-way analysis of variance followed by Tukey post hoc test (**b**, **d**, **f**, and **g**); **p < 0.01, ***p < 0.001

C3/C3aR activation can induce impairments in blood-cerebrospinal fluid barrier (BCSFB) in the choroid plexus, which has been implicated in cognitive decline [21, 29]. Thus, we assessed choroidal BCSFB dysfunction on postoperative day 1. First, we examined changes in choroidal C3 level by surgery. Compared to naive, there was a significant elevation of C3 level in the choroid plexus in the surgery group (surgery vs. naïve: *p* < 0.001; Fig. 5a, b) but not sham group (sham vs. naïve: *p* > 0.05; Fig. 5a, b). Next, we evaluated changes in IgG, ICAM-1, and VCAM-1 immunostainings in the choroidal BCSFB. Orthopedic surgery upregulated levels of IgG, ICAM-1, and VCAM-1 (Fig. 5c–g) compared to sham mice. In contrast, C3aR blockade decreased the choroidal levels of these markers (Fig. 5c–g) after surgery, suggesting surgery impairs BCSFB in the choroid plexus.

### C3aR blockade improves cognition after orthopedic surgery

**Fig. 6:**
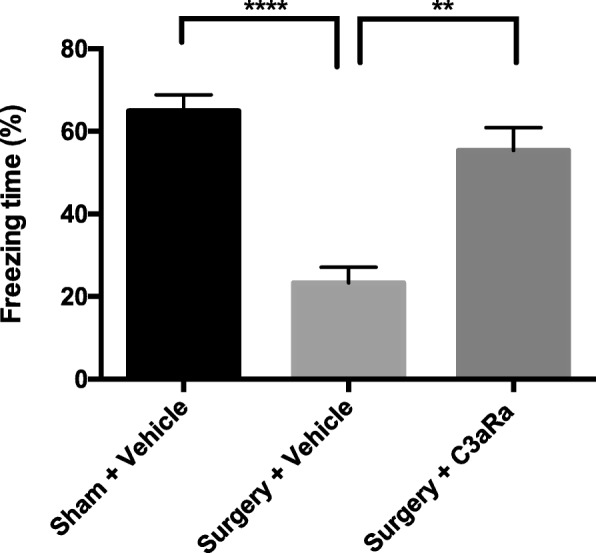
Hippocampal-dependent memory dysfunction after orthopedic surgery is ameliorated by C3aR blockade. Quantification of the percentage of freezing behavior during the context test on postoperative day 3. Data analyses were performed using one-way analysis of variance followed by Tukey post hoc test. **p < 0.01, ****p < 0.0001

Abnormal activation of C3/C3aR signaling has been correlated with memory deficits [17]. To evaluate the impact on cognition, we assessed the effects of C3aR blockade on freezing behavior using trace fear conditioning. On postoperative day 3, mice that underwent surgery showed lower freezing to the context compared to sham mice (surgery + vehicle vs. sham + vehicle: 23.29% ± 3.80 vs. 64.96% ± 3.89, *p* < 0.0001; Fig. 6). Notably, treatment with C3aR antagonist significantly improved freezing behavior (surgery + C3aRa vs. surgery + vehicle: 55.38% ± 5.51 vs. 23.29% ± 3.80, *p* < 0.01; Fig. 6).

### Exogenous administration of C3a triggers PND-like features

**Fig. 7:**
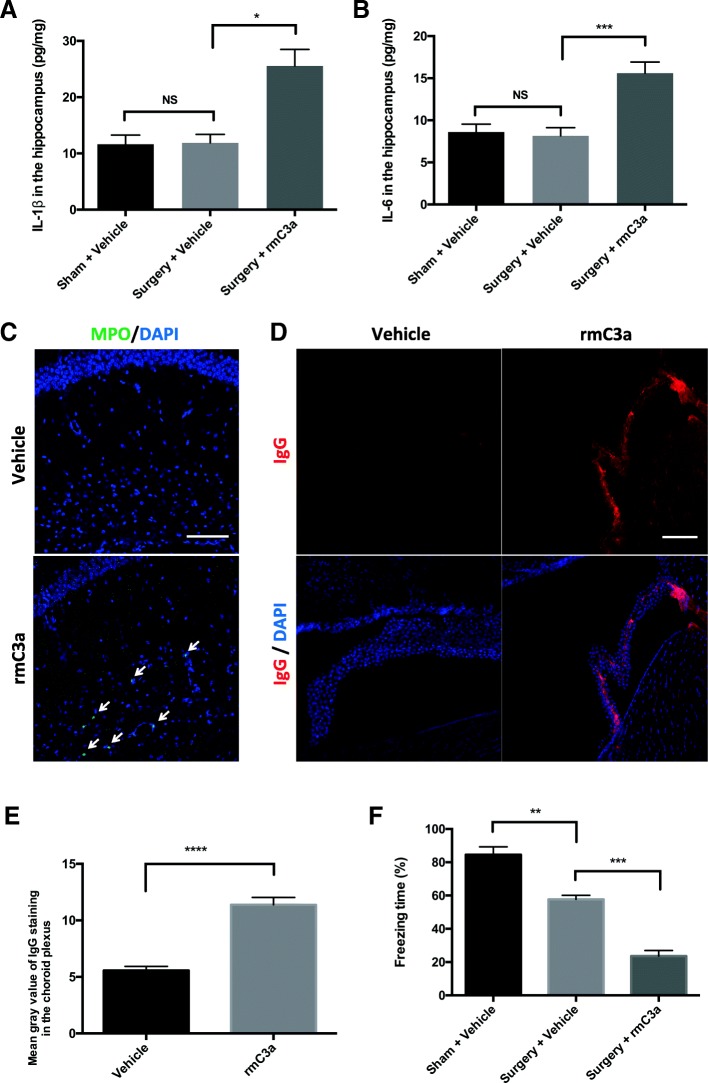
Pharmacological activation of C3aR exacerbated PND-like pathology. ELISA was used to quantify hippocampal IL-1β (**a**) and IL-6 (**b**) on postoperative day 3 in three groups: sham + vehicle, surgery + vehicle, and surgery + rmC3a (one-way analysis of variance followed by Student-Newman-Keuls test; *n* = 5). **c** Representative images of MPO staining in the hippocampus of vehicle- and rmC3a-treated mice; white arrows indicate MPO^+^ cells. **d** Representative images of IgG labeling in the choroid plexus of vehicle- and rmC3a-treated mice. Nuclear counterstaining with DAPI (blue) (**c**, **d**). **e** Quantification of IgG fluorescence intensity (unpaired Student’s *t* test; *n* = 5). **f** Quantification of the percentage of freezing time during the context on postoperative day 1 (one-way analysis of variance followed by Tukey post hoc test). Scale bar = 100 μm (**a** and **c**). *p < 0.05, **p < 0.01, ***p < 0.001, ****p < 0.0001

We further evaluated the role of C3/C3aR signaling in PND by administering exogenous C3a. First, we investigated whether rmC3a treatment caused neuroinflammation. 3 days after surgery, both IL-1β and IL-6 in the hippocampus were decreased to sham levels (surgery + vehicle vs sham + vehicle: *p* > 0.05 for both IL-1β and IL-6; Fig. 7a, b). However, mice exposed to rmC3a showed prolonged upregulation of hippocampal IL-1β (surgery + rmC3a vs. surgery + vehicle: 25.56 ± 6.56 pg/mg vs. 11.88 ± 1.51 pg/mg, *p* < 0.05; Fig. 7a) and IL-6 (surgery + rmC3a vs. surgery + vehicle: 15.60 ± 1.32 pg/mg vs. 8.17 ± 0.97 pg/mg, *p* < 0.001; Fig. 7b). Furthermore, MPO^+^ neutrophils were detected in the hippocampus in rmC3a-treated naïve mice (Fig. 7c), overall suggesting exogenous C3a prolongs surgery-induced neuroinflammation in this 2-hit model. We then examined the impact of exogenous C3a on choroidal BCSFB. Naïve mice treated with rmC3a showed evident BCSFB disruption with elevated IgG deposition in the choroid plexus compared to vehicle-treated mice (rmC3a vs. vehicle: *p* < 0.0001; Fig. 7d, e). Finally, we evaluated the effect of exogenous C3a on memory. Orthopedic surgery induced a reduction of freezing behavior on postoperative day 1 compared to sham (surgery + vehicle vs sham + vehicle: 57.73% ± 2.41 vs. 84.54% ± 4.76, *p* < 0.01; Fig. 7f). This reduction was exacerbated when mice were subjected to both surgery and exogenous C3a (surgery + rmC3a vs. surgery + vehicle: 23.64% ± 3.30 vs. 57.73% ± 2.41, *p* < 0.001; Fig. 7f).

## Discussion

Complement cascade, specially C3/C3aR signaling activation, underlies several neurological conditions featuring CNS inflammation, synapse dysfunction, choroidal BCSFB permeability changes, and cognitive decline in different models of neurological disorders [16–21]. In the present study, we used biochemical, immunohistochemical, and behavioral assays to demonstrate that C3/C3aR signaling contributes to surgery-induced neuroinflammation, synapse loss, choroidal BCSFB dysfunction, and memory deficits in a mouse model of PND-like behavior.

### Orthopedic surgery and C3/C3aR signaling in the hippocampus

Under pathological conditions, excessive hippocampal C3 deposition has been implicated in the development of many neurological disorders [13, 14]. In the current model, we found an early and significant elevation of hippocampal C3 levels after orthopedic surgery, supporting the involvement of complement activation in the pathophysiology of PND. Notably, we found C3 was primarily expressed in astrocytes, but not in microglia or neurons. Previous studies have shown that potent inducers of C3 synthesis are IL-1β for astrocytes [30] and tumor necrosis factor-α (TNF-α) for microglia ex vivo [31] while the former but not the latter is elevated in the hippocampus after orthopedic surgery [32, 33]. Thus, astrocytes might be major source of C3 in the current model, which needs further interrogation by future studies.

Microglia are the main cell type that express C3aR in the CNS [19]. Microglial C3aR has been reported to mediate neuroinflammation, β-amyloid pathology, and synapse loss [19, 20]. Here, we showed that C3aR expression in microglia in the hippocampus increased at 1 day after surgery, suggesting orthopedic surgery activates microglial C3aR. These findings also implicate the potential crosstalk between astrocytes and microglia through C3/C3aR signaling. In fact, activated microglia are potent inducers of reactive A1 astrocytes following secretion of pro-inflammatory cytokines like TNF-α, IL-1β, and C1q [34]. This may provide additional targets for upstream modulation of complement signaling and glia activation in PND.

### C3aR activation and neuroinflammation

Postoperative neuroinflammation involves elevation of pro-inflammatory cytokines [32, 33], neutrophil infiltration [35], and glia activation [23]. In the hippocampus, pro-inflammatory cytokines are acutely, yet transiently, elevated after surgery and return to baselines by postoperative day 3 [33, 36]. This increase is partially due to local de novo synthesis, with higher mRNA and protein levels of IL-1β and IL-6 in the hippocampus [36]. In a mouse model of Alzheimer’s disease, C3 knockout reduced pro-inflammatory cytokine expressions in the brain, indicating a key role for C3 and/or its downstream signaling in cytokine productions. Here, we found that C3aR blockade also reduced IL-1β and IL-6 levels already at 6 h while C3aR activation by both exogenous C3a and surgical insult prolonged the IL-1β and IL-6 upregulation at 3 days after orthopedic surgery. Of note, activated microglia are one of the primary sources of pro-inflammatory cytokines in the inflamed CNS [37]. Thus, our findings suggest that C3aR activation contributes to hippocampal IL-1β and IL-6 elevations after orthopedic surgery, possibly through microglial activation, although impaired endothelial function with infiltration of peripheral immune cells is also observed following surgery [5, 36, 38]. In fact, neuroinflammation can be also triggered by peripheral factors, including immune cells like macrophages and neutrophil infiltration [39], which is associated with PND [35]. Microgliosis has long been implicated in PND, although the underlying mechanisms for microglial activation remain unclear [3]. We found microglia activation at 1 day after surgery was effectively reduced by pretreatment with C3aR antagonist. Consistent with our finding, previous work showed that C3aR blockade attenuates hippocampal microgliosis in a mouse model of Alzheimer’s disease [19]. Notably, C3aR is expressed in brain endothelial cells [40] and C3/C3aR signaling has been reported to mediate neutrophil infiltration into the brain following lipopolysaccharide administration [41]. Under neuroinflammatory conditions, increased expression of adhesion molecules in activated endothelial cells can mediate the recruitment of neutrophils into the brain parenchyma [42]. Here, we showed that C3aR activation after orthopedic surgery contributes to neutrophil infiltration in the hippocampus, possibly by modifying adhesion molecule expressions in hippocampal endothelium and disrupting BCSFB function.

### Complement activation and synapse loss

Microglia-mediated synapse loss has been implicated in the pathophysiology of PND [8]. Upregulation of CD68 immunoreactivity indicates enhanced phagocytic activity in microglia and can be related to increased synapse engulfment [16, 28]. In the current study, we showed a linear relationship between loss of synaptic proteins and CD68 immunoreactivity, suggesting orthopedic surgery increases microglial phagocytosis of hippocampal synapses. Furthermore, surgery-induced microglial CD68 upregulation and synapse loss were both attenuated by C3aR blockade. Previous work investigating the role of complement-microglia axis in synapse elimination demonstrated both C3 and C3aR knockout were protected by synapse loss after West Nile virus infection, implicating a pivotal role for C3/C3aR signaling in microglia-mediated synapse elimination [20]. Although we do not have direct evidence showing surgery-activated microglia to engulf synapses in the present study, our findings provide initial evidence that C3aR activation contributes to synapses loss after orthopedic surgery.

### C3aR signaling and choroidal BCSFB dysfunction

The choroid plexus consists of an organized structure of epithelial cells regulating blood-CSF interactions and promoting the clearance of noxious molecules [43]. Choroidal BCSFB dysfunction underlies leukocyte infiltration, reduced neurogenesis, and cognitive decline in many neurological diseases [43]. In the current study, we found orthopedic surgery significantly induced C3 deposition in the choroid plexus. Furthermore, BCSFB permeability, as assessed by IgG deposition, was increased after surgery and normalized by C3aR blockade. Pharmacological activation of C3aR in naïve mice mimicked surgery-induced IgG elevation in the choroidal BCSFB, suggesting C3/C3aR is involved in BCSFB disruption after surgery.

Choroidal BCSFB is a common entry point for leukocyte infiltration into the brain [43] and may allow systemic factors to enter the CNS and contribute to neuroinflammation in PND. Indeed, peripheral neutrophils, macrophages, and T cells can migrate into the brain through BCSFB as shown in animal models of stroke, traumatic brain injury, and Alzheimer’s disease [44]. The choroid plexus also constitutively expresses markers of epithelial cells such as ICAM-1 and VCAM-1 [45]. Upon activation, they mediate leukocyte infiltration into the CNS [46]. Here, we found that ICAM-1 and VCAM-1 were markedly increased in the choroid plexus postoperatively, suggesting surgery activates choroidal epithelium. This surgery-induced epithelial activation may further contribute to neutrophil and macrophage infiltration [5, 36].

### Memory deficits and C3 signaling modulation

Orthopedic surgery has been increasingly shown to impair memory processes in rodent models [5, 32, 36, 47], and it commonly affects the recovery of patients after procedures like hip joint replacement [48]. Here, we show that surgery-induced cognitive impairment can be attenuated by prophylactic C3aR blockade and, conversely, we could exacerbate cognition by exogenous C3a administration. The effects of C3aR signaling manipulation on cognition may be due to several factors including changes in neuroinflammation, synapse numbers, and choroidal BCSFB function as reported in this study. Additional cognitive testing may provide insights into the role of complement signaling in PND. Notably, a preliminary clinical trial in cardiac surgical patients found no significant improvement in global cognition following administration of a monoclonal antibody directed against the C5 complement component, although some improvements were observed in the visuo-spatial domain testing [49]. Future studies should further evaluate the components of the complement cascade as well as the timing for possible interventions.

Some limitations of our study must be pointed out. First, we could not exclude the possibility that the therapeutic effects of C3aRa were partially due to its systemic effects as macrophages, which also expressed C3aR [50], are recruited into the hippocampus after surgery to exacerbate neuroinflammation and cognitive dysfunction [5, 36]. Second, further studies are needed to understand the neuro-glia crosstalk in this model and the contribution of astrocytic C3-microglial C3aR signaling on synaptic pruning and postoperative neuroinflammation. Third, we only studied the relatively short-term effects of C3/C3aR signaling manipulations on neuroinflammation and cognition. Although this may inform about the pathogenesis of acute cognitive deficits, like postoperative delirium, longer-lasting assessments combined with more clinically relevant models (i.e., aging, diabetes) should be sought in future studies.

## Conclusions

In summary, activation of C3/C3aR signaling after orthopedic surgery contributes to postoperative neuroinflammation, synapse loss, BCSFB dysfunction, and ensuing cognitive impairment. C3aR blockade may represent a promising target for PND and future studies should further evaluate the role of complement signaling after major surgery.

## Additional file

Additional file 1: Representative images of C3/NeuN double immunostaining show no detectable C3 in hippocampal neurons on day 1. (PDF 3770 kb)

## Abbreviations

ANOVA: One-way analysis of variance
BCA: Bicinchoninic acid
BCSFB: Blood-cerebrospinal fluid barrier
C3aR: C3a receptor
C3aRa: C3aR antagonist
CNS: Central nervous system
DMSO: Dimethyl sulfoxide
ELISA: Enzyme-linked immunosorbent assay
GFAP: Glial fibrillary acidic protein
HRP: Horseradish peroxidase
IBA1: Ionized calcium-binding adapter molecule 1
ICAM-1: Intercellular adhesion molecule-1
IgG: Immunoglobulin G
IL-1β: Interleukin-1β
IL-6: Interleukin-6
MPO: Myeloperoxidase
PBS: Phosphate-buffered saline
PFA: Paraformaldehyde
PND: Perioperative neurocognitive disorders
PSD-95: Postsynaptic density protein 95
rmC3a: Recombinant mouse C3a
RT: Room temperature
SYP: Synaptophysin
TNF- α: Tumor necrosis factor-α
VCAM-1: Vascular cell adhesion molecule-1
