# Supplementary figures and images for: Complement activation contributes to perioperative neurocognitive disorders in mice
Source: J Neuroinflammation. 2018 Sep 4;15:254. doi: 10.1186/s12974-018-1292-4 (PMC6123969; doi:10.1186/s12974-018-1292-4)

**C3**

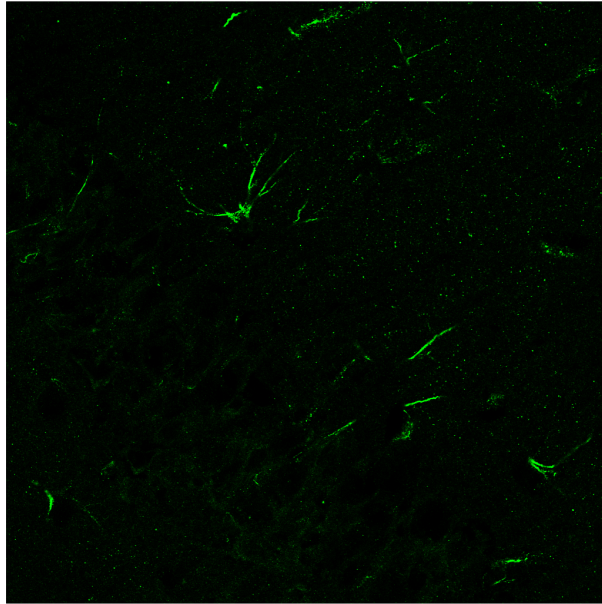

**NeuN**

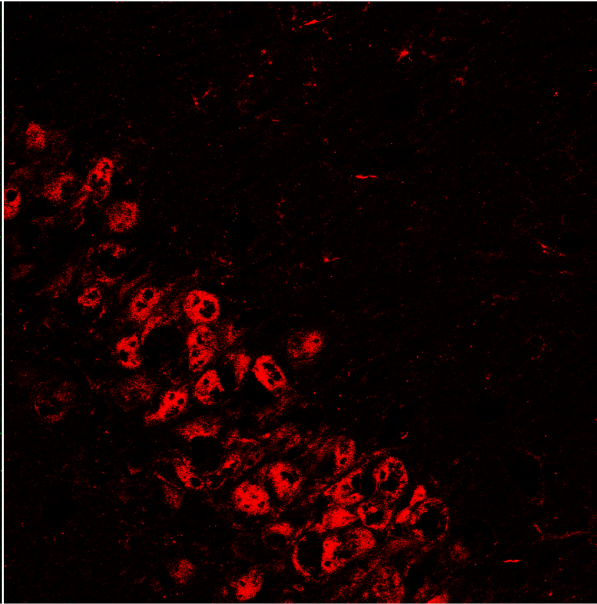

**Merge**

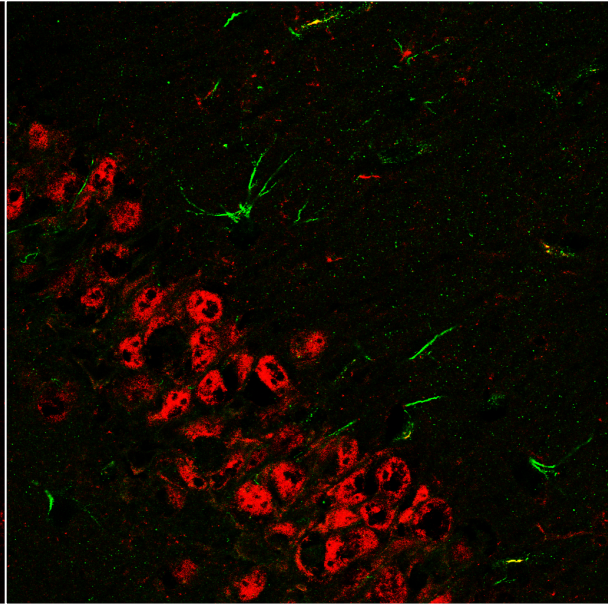

Supplement: Supplementary file 1 — Representative images of C3/NeuN double immunostaining show no detectable C3 in hippocampal neurons on day 1. (PDF 3770 kb) [file 12974_2018_1292_MOESM1_ESM.pdf]
